# Digitalis

**Published:** 1885-08

**Authors:** 


### DOMESTIC CORRESPONDENCE.

---

To THE EDITORS OF THE *Chicago Medical Journal and Examiner.*

*Gentlemen:*

In a late visit to an old New England town, I came across this letter, which you see takes us back a hundred years.

It was in possession of Dr. W. G. Perry, whose family has been known in the medical profession since 1815. His father, now at the advanced age of ninety-six, is the oldest living graduate of Harvard University, and has practiced in Exeter, New Hampshire, for more than sixty years.

Thinking that these words, coming to us from other generations, about one of our most valued medicines, might not be uninteresting to your readers, I place them at your disposal.

Respectfully,

CHAS. GILMAN SMITH, M.D.

125 State Street.

---

DIGITALIS.

BIRMINGHAM, 27th, Oct., 1786.

*Sir:*

Your letter of February 9th, arrived here in due time, and the seed of the Digitalis purpurea which accompanies this, will show that I have not been inattentive to your request. I send much more than will be necessary for your own use, in the hope that you will distribute it into other provinces, and you can transmit some to my friend S. Jones in Virginia for the same purpose. Mr. Cutler, in the memoirs of the American Academy of Arts and Sciences, printed at Boston 1785 and now before me, gives in his account of indigenous American vegetables, page 465, the Linnæan character of the Digitalis purpurea and supposes it a native of that part of America; but I have reason to believe that his species is not the *purpurea.* Perhaps you may have an opportunity of sending him a few of these seeds and please to mention my suspicion to him.

It has been remarked that in cold countries the sedentary and the intemperate are particularly the victims of disease, and the diseases which attack them are I think mostly such as arise from debility. If the kind of them you mention be really more unwholesome than that we have from the West Indies, it must be from some combination with the ardent spirit, though I am inclined to suspect that the intemperate use of Spirits, as such, will occasion the disorders you mention. If, however, the New Rum distilled from the molasses be, as you believe, more deleterious than such as we use, and that deleterious quality abates by keeping, we must suppose its melioration is caused by the separation of the hurtful impregnation. The noxious part I believe to be sometimes Lead, and sometimes an essential oil. The oil escapes in time, and I know the Lead will be precipitated by the astringent extract the spirit gets from the casks, but I never have been satisfied whether the Lead is held in solution by an oil or an acid; probably by the latter, for we find the corks of Rum bottles become rotten. If you suppose, with me, either with or without glandular disease in the abdomen, may be caused by want of action in the absorbent vessels and too great laxity in the exhalants you will readily conceive how the inaction must follow the repeated use of strong stimulants, such as ardent spirits, essential oils, or both combined. Nor will you be at a loss to judge why a cold climate and an inactive life should aid the formation of the disease. I should attempt in your dropsies to evacuate the water by the Digitalis and to prevent its repeated aggregation by frictions, exercise, flannel shirts, and other warm clothing, and should also call in the aid of aromatics, steel, and sometimes mercury.

I am more and more convinced that the Digitalis, under a judicious management, is one of the mildest and safest medicines we have, as well as one of the most efficacious. It is, I believe, *never* necessary to create nausea or any other disturbance in the system. I never now use more than ʒi fʒi to f℥ss of infusion and in substance rarely exceed 3 grains in 24 hours.

An account of your tryals with this medicine will be highly grateful to me, and fully recompense any trouble you have given me.

The experiments in the freezing of Quick silver were extremely well conducted, and correspond sufficiently with the most accurate accounts we have had from other quarters. I read them to a Society of Philosophic friends in the place, and they met the approbation of all. Reason and Philosophy are making rapid strides in every quarter of the globe to emancipate and to enlighten mankind. We cure our Intermittents of every kind by a solution of white Arsenic in water, 1 grain to ʒi; dose, 25 or 30 drops, 3 times a day, in 6 or 8 ounces of Gruel or Barley water. In or out of the paroxysms we go on, until the disease is stopped. The cure is then to be finished with the usual tonics. Under proper management it produces no sensible effects.

I must beg you to accept my grateful thanks for the opinion you are pleased to entertain of me, and to believe me that no one is more happy to promote the spread of science than your obliged and very,

Obdt! Servt!

W. WITHERING.

The new Botan. arrangement will not be out sooner than the beginning of next summer. Digitalis has cured two other cases of insanity in this neighborhood and 3 cases of Haemoptoe. The latter were of the kind attended with a quick bounding pulse, and I directed the medicine from the quality I knew it possessed of abating the action of the heart.

